# [Retracted] Na^+^/K^+^-ATPase DR region-specific antibody protects U251 cells against hypoxia reperfusion-induced injury via the PI3K/AKT and ERK pathways

**DOI:** 10.3892/mmr.2026.13853

**Published:** 2026-03-23

**Authors:** Huilin Gong, Pengbiao Lü, Jiangwei Zhang, Dandong Li, Jin Zheng, Jinning Song

Mol Med Rep 16: 7901–7906, 2017; DOI: 10.3892/mmr.2017.7622

Following the publication of this paper, it was drawn to the Editor's attention by a concerned reader that several of the flow cytometric plots featured in Fig. 2C on p. 7904 contained apparently repeating patterns of dots, both vertically and horizontally, which were markedly more similar than might have been expected.

After making an internal assessment of these data in the Editorial Office, he Editor of *Molecular Medicine Reports* has decided that this paper should be retracted from the journal on account of a lack of confidence in the presented data. The authors were asked for an explanation to account for these concerns, but the Editorial Office did not receive a reply. The Editor apologizes to the readership for any inconvenience caused.

